# Relationship between job satisfaction and work engagement in Chinese kindergarten teachers: Vocational delay of gratification as a mediator

**DOI:** 10.3389/fpsyg.2023.1114519

**Published:** 2023-02-23

**Authors:** Lingling Zang, Yuanchun Feng

**Affiliations:** ^1^Faulty of Education, Henan University, Kaifeng, China; ^2^School of Music and Dance, Zhengzhou University of Science and Technology, Zhengzhou, China

**Keywords:** work engagement, job satisfaction, vocational delay of gratification, kindergarten teacher, mediating effect

## Abstract

Job satisfaction as a positive job-related emotional state affects teachers’ work engagement. This study explored the relationship between job satisfaction and work engagement in kindergarten teachers and the mediating role of vocational delay of gratification. Six hundred and eleven kindergarten teachers from China were surveyed with the Job Satisfaction Scale, Work Engagement Scale, and Vocational Delay of Gratification Scale. Results showed that kindergarten teachers’ job satisfaction and vocational delay of gratification were positively correlated with work engagement, and vocational delay of gratification played a mediating role between job satisfaction and work engagement. Results of the relationship among job satisfaction, vocational delay of gratification, and work engagement were discussed considering the background of Chinese culture.

## Introduction

With the development of positive psychology, work engagement has received increasing attention from researchers and educators (Macey and Schneider, 2008; Chen et al., 2018). Teachers’ work engagement not only affects their own performance and professional development (Li, 2019; Xu and Yin, 2020; Liu and Wen, 2022), but also plays an important role in students’ academic achievement and physical and mental development (Penttinen et al., 2020; Vujčić et al., 2022). Kindergarten teachers are facing the dual tasks of taking care of and educating children aged from 3 to 6, which means more time and energy are required from teachers. In China, kindergarten teachers are generally facing the reality of having low income and high work stress (Hu and Hu, 2018; Gong et al., 2020), making them more likely to have negative emotional experiences that affect work engagement. Therefore, exploring the influencing factors and mechanism of work engagement in kindergarten teachers is helpful to reduce the negative emotional experiences and promote the work engagement, which will ultimately guarantee the quality of teaching and education.

Work engagement is a positive, fulfilling state of mind associated with work, characterized by vigor (high levels of energy and mental resilience while working), dedication (a sense of significance, enthusiasm, inspiration, pride, and challenge), and absorption (a happy state of complete immersion in work) (Schaufeli et al., 2002). It has been proved that job satisfaction is an important factor in predicting work engagement (Liu, 2015; Yalabik et al., 2017), but this relationship is rarely discussed among kindergarten teachers. The relationship and mechanism of the two needs to be further explored. Meanwhile, as a choice orientation that is willing to give up immediate satisfaction for valuable and long-term goals (Liu and Huang, 2013), vocational delay of gratification is related to job satisfaction (Liu et al., 2007; Wen, 2015), and also have an impact on work engagement (Wang, 2009; Shi et al., 2021). It can be seen that there is a close relationship among job satisfaction, vocational delay of gratification, and work engagement. However, there is still no research on whether and how kindergarten teachers’ job satisfaction and vocational delay of gratification are related to their work engagement. Therefore, this study focuses on Chinese kindergarten teachers to explore the relationship and mechanism among job satisfaction, vocational delay of gratification, and work engagement.

## Literature review and hypothesis development

### Job satisfaction and work engagement

Job satisfaction is a pleasurable emotional state that arises from the evaluation of the job as achieving or facilitating the achievement of job values (Locke, 1969). Social exchange theory suggests that when individuals can get a pleasant experience from work, i.e., high satisfaction, he or she will increase work commitment to reward the organization (Blau, 1964). Research from Yalabik et al. (2017) showed that there is a significant correlation between job satisfaction and work engagement and the “satisfaction with current work” among all the job satisfaction facets is the key driver of all dimensions of work engagement. A study of 420 corporate employees also found a significant correlation between job satisfaction and work engagement (Li, 2020). Teachers’ job satisfaction is the degree of a positive emotional orientation resulting from teachers when they consider the various aspects related to their job after they actually engage in teaching (Wang, 2005). From these definitions, it is clear that teachers’ job satisfaction is a positive emotional experience based on cognition, and positive emotional experience that can significantly predict their work engagement (Høigaard et al., 2012; Li et al., 2013; Wu et al., 2020; Shi et al., 2021). Studies show that teachers’ job satisfaction has a direct impact on their work engagement, which means that the more satisfied teachers are with their jobs, the more they are able to engage in their work in a positive way (Dina et al., 2016; Pepe et al., 2019; Butakor et al., 2021). Therefore, improving teachers’ job satisfaction can effectively promote their work engagement.

### Vocational delay of gratification as a mediator

Vocational delay of gratification is the application of delayed gratification in organizational psychology. Delayed gratification is a concept put forward by Mischel and Underwood (1974), which refers to the individual’s choice orientation to actively forgo immediate gratification for a more valuable long-term outcome, and the self-control demonstrated in waiting. Reynolds and Schiffbauer (2005) defined vocational of delayed gratification as the ability to maintain the choice that is conducive to achieving long-term career goals and achievements even though there is an immediate opportunity for short-term gratification. Liu et al. (2007) believe that vocational of delayed gratification is a choice orientation that is willing to give up immediate gratification opportunities that are not conducive to current work in order to achieve a series of valuable long-term results. In general, vocational delayed of gratification is a decision-making orientation to give up immediate gratification in order to achieve long-term career goals. Unlike delayed gratification as a stable personality trait, vocational delay of gratification is more of a behavioral tendency to achieve a goal, which is positively related to job satisfaction (Mohsin and Ayub, 2014; Wen, 2015; Xu and Yin, 2020). But there is little research on the relationship between vocational delay of gratification and job satisfaction among teachers. Vocational delay of gratification can be considered an important individual resource as it contributes to the achievement of individual goals (Halbesleben et al., 2014). According to the conservation of resources theory, valuable individual resources help individuals to overcome the stress and burnout caused by the loss of resources and promote their work engagement by generating incremental own resources (Hobfoll et al., 2018). It has also been shown that there is a significant correlation between vocational delay of gratification and work engagement and that vocational delay of gratification is a significant predictor of work engagement (Wo, 2013; Zhao, 2015). Shi et al. (2021) took 1,176 rural teachers in poor areas as an example to study the relationship between vocational delay of gratification and work engagement and results showed that there was a significantly positively correlation between them. As a result, it can be seen that vocational delay of gratification is influenced by job satisfaction and can directly affect work engagement. Moreover, the influence mechanism of job satisfaction on work engagement can be well revealed from the perspective of vocational delayed of gratification.

To sum up, the existing research provides a preliminary understanding of the relationship among job satisfaction, vocational delay of gratification, and work engagement. This study attempts to explore the relationship between job satisfaction and work engagement of kindergarten teachers in China, and whether vocational delay of gratification plays a mediating role between the two. Therefore, according to social exchange theory, conservation of resources theory, and recent studies, the following hypotheses are proposed.

*H1*: There is a significant correlation between job satisfaction, vocational delay of gratification, and work engagement in Chinese kindergarten teachers.

*H2*: Vocational delay of gratification mediates the relationship between job satisfaction and work engagement in Chinese kindergarten teachers.

## Research methodology

### Research approach

Questionnaire was used in this study, which is the most commonly used method in education research to quickly collect data from a target population. There are two reasons for adopting this research approach. First, the study focused on the relationship among job satisfaction, vocational delay of gratification, and work engagement, and the questionnaire is helpful to measure variables. Second, a questionnaire survey is convenient and practical as it can collect a large number of sample data in a short time. At the same time, questionnaire is conducted anonymously, which can reduce respondents’ concerns to obtain more real information.

### Participants and procedures

According to empirical estimates of sample sizes needed for 0.8 power (Fritz and Mackinnon, 2007), the minimum sample sizes for this study is 462 participants. Considering possible error in variables measurement, larger sample sizes will be needed. After estimating the overall sample size, a three-stage stratified random sampling design was used to investigate kindergarten teachers in Henan Province. First of all, cities were stratified into three layers (upper, middle, and lower) in Henan province by cluster analysis based on GDP, urbanization level, and education index. Second, one city was extracted from each layer. For example, Zhengzhou represents the upper level, Luoyang represents the middle level, and Kaifeng represents the lower level. Finally, 10 kindergartens were selected in each city, taking into account the area (rural and urban) and type (public and private), and a total of 30 kindergartens were selected. All participants were professional teachers in kindergartens, excluding nurses.

Questionnaires were distributed by the combination of on-site survey and online survey, and the network survey was carried out through professional platform named “Wenjuanxing.” All questionnaires were conducted in Chinese. Prior to the investigation, permission was granted from kindergartens and the informed consent is provided to all the participants. Questionnaires were distributed to kindergarten teachers in September 2021 and were collected within 1 week. In order to ensure the data quality, participants were informed that the study was an anonymous survey and results were only for academic research, so as to reduce the psychological defense and social approval effects. A total of 693 questionnaires were sent out to 30 selected kindergartens and 646 were recovered, with a recovery rate of 93.2%. Of 646 questionnaires, 611 were valid and formed the final sample. The invalid questionnaires were eliminated following the criteria for elimination: (a) missing data; (b) a regular pattern of responses; (c) contradictory responses to relevant items (e.g., inconsistent responses to homogeneous items or consistent responses to opposing items). The demographic characteristics of the sample are shown in Table 1.

**Table 1:** Demographic information of the sample (*n* = 611).

Features	*n* (%)
Area
Urban	385 (63.0)
Rural	226 (37.0)
Kindergarten type
Public	363 (59.4)
Private	248 (40.6)
Gender
Female	601 (98.4)
Male	10 (1.6)
Teaching experience
≤5 years	311 (50.9)
6–10 years	161 (26.4)
11–15 years	70 (11.5)
16–20 years	28 (4.6)
≥21 years	41 (6.7)
Education level
≤Secondary technical school	81 (13.3)
Junior college	299 (48.9)
Bachelor	228 (37.3)
Postgraduate	3 (0.5)

### Measures

#### Job satisfaction scale

Job satisfaction was measured using the Kindergarten Teacher Job Satisfaction Questionnaire compiled by Wang (2005) according to the work characteristics of Chinese kindergarten teachers. The scale includes five dimensions: current work (e.g., I am satisfied with my current work schedule), job environment (e.g., I am satisfied with the interpersonal relationships among colleagues), work remuneration (e.g., I am satisfied with the current welfare system), advanced study and promotion (e.g., I think there are many opportunities for advanced study), and director (e.g., I think director can handle disputes among teachers fairly). All items were scored on a 6-point Likert scale ranging from 1 (strongly disagree) to 6 (strongly agree), where items 12, 15, 16, and 23 will be reverse scored. Higher scores in each dimension indicate higher job satisfaction. In this study, the Cronbach’s *ɑ* is 0.95. The internal consistency of current work is 0.88, job environment is 0.81, work remuneration is 0.64, advanced study and promotion is 0.89, and director is 0.97.

#### Work engagement scale

Work engagement scale was used to evaluate the degree of work engagement of kindergarten teachers. The scale was compiled by Wang and Qin (2010) on the basis of the Utrecht Work Engagement Scale (UWES) developed by Schaufeli (Schaufeli et al., 2002), and combined with the actual work situation of kindergarten teachers in China. The questionnaire consists of 21 items designed to assess four dimensions of kindergarten teachers’ work engagement: work enjoyment (e.g., the worries in life quickly disappear when I come to kindergarten), work value (e.g., I find preschool education work meaningful), work responsibility (e.g., I take my work seriously), and work concentration (e.g., I reach a state of forgetfulness when I am in class). All items were scored on a 5-point Likert scale (1 = never to 5 = always). Higher scores on the four dimensions indicate higher levels of work engagement among kindergarten teachers. In our sample, the Cronbach’s *ɑ* was 0.93. The internal consistency of four dimensions was 0.81 for work enjoyment, 0.83 for work value, 0.84 for work responsibility, and 0.78 for work concentration.

#### Vocational delay of gratification scale

Vocational delay of gratification in this study was measured using the Vocational Delay of Gratification Questionnaire developed by Liu et al. (2007), which consists of eight items to measure two dimensions: job delayed gratification (e.g., I often work late into the night to better complete my work) and career delayed gratification (for example, as long as there is room for further development, it does not matter to start small or start all over again). The higher the scores, the higher the vocational delay of gratification. The scale has been used in the survey of kindergarten teachers with good reliability and validity (Shi et al., 2021). All items were obtained on 4-point Likert scale, ranging from 1 (very non-conforming) to 4 (very conforming). In this scale, the Cronbach’s *ɑ* was 0.77. The internal consistency of job delay satisfaction was 0.64, and career delay satisfaction was 0.71.

### Data analysis

SPSS 26.0 was used in this study to conduct descriptive statistics, correlation analysis, regression analysis of kindergarten teachers’ job satisfaction, vocational delay of gratification, and work engagement. Descriptive statistics were used to analyze the scores of the three variables and their dimensions. Correlation analysis was used to analyze the pairwise correlation among three variables. Regression analysis was used to analyze the influence of independent variable on dependent variable, that is, to what extent independent variables can explain the change of dependent variables. The Bias-corrected Bootstrap method of deviation correction was used to test the mediating effect of vocational delay of gratification between job satisfaction and work engagement. Bootstrap method is a method of repeated sampling from a sample, provided that the sample is representative of the total, i.e., the sample is repeatedly sampled from a given sample in a put-back manner to produce many samples (Preacher and Hayes, 2008). The sample set for this study is 1,000, which means that estimates of the product of 1,000 coefficients can be obtained. They are then sorted from smallest to largest values, where the 2.5th and 97.5th percentile constitute a confidence interval (CI) with 95%, whereby the test can be performed. If the confidence interval does not include 0, the product of coefficient is significant, i.e., the mediating effect is significant.

## Results

### Descriptives and comparisons among variables

The means of kindergarten teachers’ job satisfaction, work engagement, and vocational delay of gratification were 4.47, 3.91, and 3.11, respectively, which were all higher than theoretical median values. In terms of job satisfaction, there were no significant differences in area, type, and education level, but significant differences in teaching experience. There were significant differences in teaching experience and educational level for work engagement. As for vocational delay of gratification, there were significant differences in area and teaching experience, but no significant differences in other aspects.

### Correlation analysis of job satisfaction, vocational delay of gratification, and work engagement

Table 2 shows the correlation analysis results among three variables. Kindergarten teachers’ job satisfaction was positively related to work engagement (*r* = 0.625, *p* < 0.001) and vocational delay of gratification (*r* = 0.475, *p* < 0.001). The correlation between current work in job satisfaction and work engagement is higher than the other four dimensions (current work, *r* = 0.684, *p* < 0.001; job environment, *r* = 0.559, *p* < 0.001; advanced study and promotion, *r* = 0.416, *p* < 0.001; work remuneration, *r* = 0.409, *p* < 0.001; director, *r* = 0.558, *p* < 0.001). Vocational delay of gratification was positively correlated with work engagement (*r* = 0.494, *p* < 0.001). Thus, H1 was supported.

**Table 2:** Correlation analysis among variables.

Variables	1	2	3	4	5	6	7	8
1. JS	–							
2. CW	0.83***	–						
3. JE	0.86***	0.74***	–					
4. ASP	0.79***	0.55***	0.66***	–				
5. WR	0.83***	0.53***	0.60***	0.65***	–			
6. DR	0.88***	0.69***	0.69***	0.59***	0.62***	–		
7. VDG	0.48***	0.54***	0.45***	0.32***	0.30***	0.41***	–	
8. WE	0.63***	0.68***	0.60***	0.42***	0.41***	0.56***	0.49***	–
Mean	4.47	5.02	4.59	3.87	3.82	4.72	3.11	3.91
SD	0.77	0.72	0.81	0.92	1.11	1.05	0.45	0.61
Cronbach’s *ɑ*	0.95	0.88	0.81	0.64	0.89	0.97	0.93	0.77

JS, job satisfaction; CW, current work; JE, job environment; ASP, advanced study and promotion; WR, work remuneration; DR, director; VDG, vocational delay of gratification; WE, work engagement; ***p < 0.001.

### Mediating effect analysis

Table 3 shows the regression analysis results of the relationship between job satisfaction and work engagement, in which area, kindergarten type, gender, teaching experience, and educational level are control variables. Results showed that job satisfaction positively predicted vocational delay of gratification (*β* = 0.29, *p* < 0.001) and work engagement (*β* = 0.48, *p* < 0.001). When vocational delay of gratification was included in the regression equation, the direct effect value of job satisfaction on work engagement is significantly reduced (*β* = 0.36, *p* < 0.001). These results indicate that vocational delay of gratification has a significant mediating effect on the relationship between job satisfaction and work engagement.

**Table 3:** Regression analysis of the relationship among variables.

	Model 1	Model 2	Model 3	Model 4	Model 5
Constant term	3.16***	1.59***	4.15***	1.61***	1.00**
Control variables
Areas	0.10*	0.15***	−0.03	0.05	−0.01
Kindergarten type	−0.05	−0.02	0.06	0.10**	0.11**
Gender	−0.01	0.03	−0.08	−0.03	−0.04
Teaching experience	−0.03*	−0.06***	0.11***	0.07***	0.09***
Educational level	−0.03	0.03	−0.15***	−0.05	−0.07*
Independent variables
JS		0.29***		0.48***	0.36***
Mediate variables
VDG					0.38***
*R* ^2^	0.03	0.27	0.07	0.42	0.48
*F*	3.07**	200.42***	8.73***	369.48***	239.63***

All variables in these models have been standardized. Model 1 took VDG as dependent variable and only introduced controls variables. Model 2 took VDG as the dependent variable, and introduced JS and control variables to conduct regression analysis. Model 3 took WE as dependent variable and only introduced control variables. Model 4 took WE as dependent variable, and introduced JS and control variables to conduct regression analysis. Model 5 took WE as dependent variable and introduced JS, VDG, and control variables to make regression analysis. JS, job satisfaction; WE, work engagement; VDG, vocational delay of gratification. *p < 0.05, **p < 0.01, ***p < 0.001.

**Figure 1:**
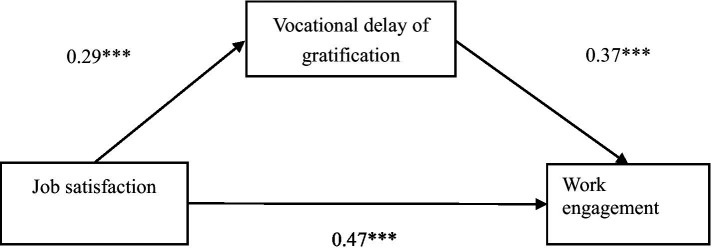
The medication model of vocational delay of gratification on the relationship between job satisfaction and work engagement. ***p < 0.001.

Bootstrap was used to test the mediating effect of vocational delay of gratification between job satisfaction and work engagement among kindergarten teachers, and the results are shown in Table 4 and Figure 1. The total effect of job satisfaction on work engagement was 0.47 (SE = 0.02, *t* = 19.10, *p* < 0.001, 95% CI [0.42, 0.52]). After the addition of vocational delay of gratification, the effect of job satisfaction on work engagement remained significant, but the value decreased to 0.36 (SE = 0.03, *t* = 13.63, *p* < 0.001, 95% CI [0.31, 0.42]). Thus, the mediating effect of vocational delay of gratification was 0.11 (SE = 0.02, 95% CI [0.08, 0.14]). This result indicates that the mediating effect is significant and vocational delay of gratification plays a partially mediating role in the influence of kindergarten teachers’ job satisfaction on work engagement, so H2 was supported.

**Table 4:** Mediating effect of vocational delay of gratification.

	Effect	SE	*t*	*p*	LLCI	ULCI
JS → WE(c)	0.47	0.02	19.10	0.000	0.42	0.52
JS → VDG(a)	0.29	0.02	13.65	0.000	0.24	0.33
VDG → WE(b)	0.37	0.05	8.13	0.000	0.28	0.46
JS → WE(c’)	0.36	0.03	13,63	0.000	0.31	0.42
JS → VDG → WE	0.11	0.02			0.08	0.14

c, the total effect of job satisfaction on work engagement; a, the effect of job satisfaction on vocational delay of gratification; b, the effect of vocational delay of gratification on work engagement; c`, the direct effect of job satisfaction on work engagement when vocational delay of gratification is included; LLCI, the lowest value of the confidence interval; ULCI, the highest value of the confidence interval; JS, job satisfaction; VDG, vocational delay of gratification; WE, work engagement.

## Discussion

The study found that teachers’ job satisfaction was above the medium level, which was the same as the existing research conclusions (Ma, 2019). The job satisfaction of teachers with more than 10 years of teaching experience is significantly higher than that of teachers with less than 5 years. This may be due to the fact that teachers with longer teaching experience have a richer working experience, professional ability has been greatly improved, and they have stable working relationships. And with the increase of working years, salaries and benefits will also increase, so the job satisfaction is higher (Lu and Luo, 2021).

Teachers` work engagement and vocational delay of gratification were all above average which was in line with previous studies (Cheng and Gao, 2019; Hou, 2020; Jia et al., 2021). As for work engagement, with the growth of teaching experience, teachers’ work engagement also increases. This increase may occur because that older teachers have fully adapted to work, with more rich experience, higher sense of achievement and satisfaction of work, and can better devote themselves to work. Teachers` work engagement with a low education background was significantly higher than that of teachers with a high education background. The reason may be that teachers with a high education background often have high work expectations, but in reality, the level of education background has no significant impact on salary and welfare. This will lead to a gap between the ideal and the reality, thus affecting their work engagement. In terms of vocational delay of gratification, the level of rural teachers is significantly higher than urban teachers. This may be because there are more job options in urban areas, and individuals are reluctant to choose delayed gratification in one occupation. The vocational delay of gratification of teachers with more than 16 years of teaching experience was significantly lower than teachers with less than 16 years of teaching experience. This may be because as they grow older, they feel that there is less room for advancement and their enthusiasm for work decreases, leading to lower levels of vocational delayed of gratification.

This study investigated the correlations among job satisfaction, work engagement, and vocational delay of gratification in kindergarten teachers. The results of the correlation analysis showed that job satisfaction was significantly positively related to work engagement, which was consistent with existing studies (Alarcon and Lyons, 2011; van der Want et al., 2019; Mérida-López and Extremera, 2020; Shuck et al., 2021; Spector, 2022). These studies suggested that individuals who are more satisfied with their jobs are more likely to be engaged in work in a more positive and active state. Of the five dimensions among job satisfaction, current work was significantly more strongly correlated with work engagement than the other four and current work significantly predicted work engagement, confirming a previous study (Ma, 2019). This shows that the work engagement of kindergarten teachers comes from their identification with current work. This may be related to the Chinese government’s emphasis on preschool education in recent years. The professional value of kindergarten teachers has been widely recognized and affirmed, and their social reputation is constantly improving (Lu and Luo, 2021). This could explain why teachers are able to maintain above moderate level of work engagement (*M* = 3.91) despite the realities of low income and high stress. At the same time, it can be seen from Table 5 that the mean of advanced study and promotion and work remuneration among job satisfaction of kindergarten teachers are the lowest, which are 3.87 and 3.82, respectively, and they are significantly positively correlated with work engagement (advanced study and promotion, *r* = 0.416, *p* < 0.001; work remuneration, *r* = 0.409, *p* < 0.001). Therefore, in order to further enhance the level of work engagement, it is necessary to consider providing more support and guarantee for teachers in terms of further education, promotion, and work remuneration.

**Table 5:** Comparison of differences in demographic variables of each variable (M ± SD).

		JS	WE	VDG
M ± SD (*N* = 611)		4.47 ± 0.77	3.91 ± 0.61	3.11 ± 0.45
Area	Urban (*N* = 385)	4.50 ± 0.82	3.89 ± 0.62	3.07 ± 0.45
	Rural (*N* = 226)	4.42 ± 0.69	3.95 ± 0.58	3.18 ± 0.46
	*t*	1.10	−1.12	−3.01**
	Comparison	–	–	Rural > Urban
Kindergarten type	Public (*N* = 363)	4.49 ± 0.83	3.88 ± 0.63	3.12 ± 0.44
	Private (*N* = 248)	4.43 ± 0.68	3.96 ± 0.57	3.10 ± 0.46
	*t*	0.99	−1.48	0.69
Teaching experience	① ≤5 years (*N* = 311)	4.40 ± 0.82	3.79 ± 0.68	3.12 ± 0.47
	② 6–10 years (*N* = 161)	4.46 ± 0.72	4.00 ± 0.49	3.10 ± 0.47
	③ 11–15 years (*N* = 70)	4.65 ± 0.76	4.10 ± 0.49	3.23 ± 0.44
	④ 16–20 years (*N* = 28)	4.46 ± 0.64	3.94 ± 0.52	2.99 ± 0.39
	⑤ ≥21 years (*N* = 41)	4.72 ± 0.61	4.17 ± 0.45	2.92 ± 0.33
	*F*	2.65*	7.92***	3.55**
	Multiple comparison	③ > ①* ⑤ > ①*	② > ①*** ③ > ①*** ⑤ > ①***	① > ⑤** ② > ⑤** ③ > ④* ③ > ⑤**
Education level	① ≤ Secondary technical school (*N* = 81)	4.63 ± 0.59	4.00 ± 0.60	3.20 ± 0.45
	②Junior college (*N* = 299)	4.50 ± 0.71	3.98 ± 0.57	3.11 ± 0.47
	③Bachelor (*N* = 228)	4.38 ± 0.90	3.80 ± 0.64	3.08 ± 0.43
	④Postgraduate (*N* = 3)	4.08 ± 0.73	3.83 ± 0.77	2.88 ± 0.00
	*F*	2.48	4.76**	1.73
	Multiple comparison	–	① > ③** ② > ③***	–

JS, job satisfaction; WE, work engagement; VDG, vocational delay of gratification; *p < 0.05, **p < 0.01, ***p < 0.001.

This study also found that there was a significant positive correlation between job satisfaction and vocational delay of gratification, which was consistent with existing research conclusions (Xie, 2014; Xu and Yin, 2020). This shows that the higher the job satisfaction, the higher the vocational delay of gratification. Vocational delay of gratification will be affected by an individual’s working environment. If a person has a high degree of matching with the organization, feels more support from the organization, and has a harmonious relationship with colleagues, he or she will show higher level of vocational delay of gratification (Zhao, 2013; Jia et al., 2021). Liu et al. (2007) showed that fair promotion and further education in organizational career management significantly predicted vocational delay of gratification, i.e., if individuals perceive that there are fair promotion opportunities and advanced study paths in the organization, they will adopt delayed satisfaction to achieve higher goals of career development. From the above, it is evident that if individuals evaluate their work environment positively, i.e., their job satisfaction is high, the level of their vocational delay of gratification is also high. It follows that the higher the job satisfaction of kindergarten teachers, the more likely they are to forego immediate gratification for more long-term goals, demonstrating higher levels of vocational delay of gratification.

This study showed that there is a significant positive correlation between vocational delay of gratification and work engagement and that vocational delay of gratification is a significant predictor of work engagement, which confirms the findings of existing studies (Xanthopoulou et al., 2009; Balay-Odao et al., 2021; Liu and Wang, 2021; Zhang et al., 2022). Studies from Shi et al. (2021) suggested that vocational delay of gratification, on the one hand, brings positive emotions and helps individuals to expand their awareness and attention, thus making them more focused and dedicated to the work at hand, and on the other hand, increases work engagement by reducing rumination and attention associated with negative emotions. From this perspective, work engagement as a positive and fulfilling work-related state of mind is significantly influenced by vocational delay of gratification. At the same time, according to the Demand-Resource Model, vocational delay of gratification as a valuable individual resource can enhance individuals’ recognition of their work and thus promote their work engagement (Demerouti et al., 2001). Under the multiple dilemmas faced by kindergarten teachers in China, teachers may need to show a higher level of vocational delayed gratification in order to better engage in their work.

The results of this study found that vocational delay of gratification partially mediated the relationship between job satisfaction and work engagement. This suggests that kindergarten teachers’ job satisfaction not only directly influences work engagement, but also can have an impact on their work engagement through vocational delay of gratification. As an important individual psychological resource, vocational delay of gratification is not only influenced by job satisfaction, but also functions as a resource preservation and expansion to help individuals better cope with job demands and thus increase work engagement (Hobfoll, 2002). This fits well with the social exchange theory (Blau, 1964). When an individual is able to derive pleasurable experiences from work, i.e., high satisfaction, he or she will increase delayed gratification such as volunteering to work overtime or taking on difficult tasks in order to increase work engagement in return for the organization. Compared with individuals with low job satisfaction, individuals with high job satisfaction voluntarily give up opportunities for immediate job satisfaction, whether it is in the current job or in the longer-term career, i.e., they are more likely to choose vocational delay of gratification (Liu and Wang, 2021; Wang et al., 2021). In other words, individuals with high level of vocational delay of gratification tend to remain actively engaged in their work in order to achieve their job goals and the increased benefits and higher career goals attached to their job goals. This suggests that kindergarten administrators and policymakers should consider both job satisfaction and vocational delay of gratification to enhance teachers’ work engagement.

### Practical implications

This study introduced vocational delay of gratification and examined the effects of job satisfaction and vocational delay of gratification on work engagement in kindergarten teachers, and found that increasing job satisfaction and vocational delay of gratification significantly increased kindergarten teachers’ work engagement.

This result provides practical implications for increasing kindergarten teachers’ work engagement. Teachers’ work engagement should be improved by increasing their job satisfaction. In Chinese context, it is important to raise salary and opportunities for advanced study and promotion to stimulate their positive emotional experience to increase work involvement. Work remuneration and advanced study and promotion are necessary working resources for individuals. Only when kindergarten teachers have sufficient working resources can they work with a positive and focused mind. The influence of job satisfaction on work engagement through vocational delay of gratification should also be taken seriously. To put it into practice is to establish clear career development pathways and fair promotion channels for kindergarten teachers. When kindergarten teachers can see the prospect of future career development from their work, they will give up the immediate satisfaction that is not conducive to the current work, and actively choose to delay the satisfaction to increase work engagement. In addition to these intrinsic incentives and rewards, attention should also be paid to the influence of the social environment on kindergarten teachers ‘job satisfaction and work engagement. Although Chinese government has taken the development of preschool education as a national strategy and has continuously increased its investment in the past 10 years (Yu, 2019), the social recognition of kindergarten teachers is still not high, and the stigmatization of male kindergarten teachers still exists. Therefore, it is also very important to enhance the social recognition of preschool education while continuously improving the internal incentives of kindergarten teachers.

### Limitation and future research directions

For this study, there are limitations that need to be stated. Firstly, only 611 kindergarten teachers in Henan Province were selected for the survey in this study, and the number of male teachers was small. The sample size was not sufficient and the selected subjects were not broad enough, which may reduce the generalizability of the results. In the future, more areas can be covered to expand the sample size, and the proportion of male teachers can be increased to enrich research results. Secondly, this study conducted a cross-sectional research design, which is not conducive to explaining causal relationships among variables. A longitudinal research design or experimental study could be adopted in the future to verify the effects of job satisfaction and vocational delay of gratification on work engagement in kindergarten teachers. Thirdly, the current study only examined the effect of kindergarten teachers’ job satisfaction on work engagement, but there are other factors affecting kindergarten teachers’ work engagement that can be further explored. Finally, the mediation model showed that vocational delay of gratification played a partial mediator between job satisfaction and work engagement, which indicated that there are other mediating factors that affect work engagement. Other mediating variables can be introduced for further exploration in the future, such as psychological capital and work-family balance, in order to more fully understand the mechanism of the effect of job satisfaction on work engagement.

## Conclusion

This study is the first to explore the relationship among job satisfaction, vocational delay of gratification, and work involvement in kindergarten teachers. The results showed the direct impact of job satisfaction on work engagement, and supported the mediating role of vocational delay of gratification between the two. Social exchange theory and resource conservation theory were used to explain the impact mechanism among three variables. These findings enrich the existing research literature, especially the understanding of the relationship between kindergarten teachers’ job satisfaction, work engagement, and vocational delay of gratification in Chinese cultural context. The other possible mediating factors between kindergarten teachers’ job satisfaction and work engagement and the antecedent variables of work engagement deserve further exploration.

## Data availability statement

The original contributions presented in the study are included in the article/Supplementary material, further inquiries can be directed to the corresponding author.

## Ethics statement

The studies involving human participants were reviewed and approved by Faculty of Education, Henan University. Participants provided their written informed consent to participate in this study.

## Author contributions

LZ and YF: research idea and research design. YF: data collection and analysis. LZ: manuscript writing. All authors contributed to the article and approved the submitted version.

## Funding

This study was funded by Henan Province key R&D and Promotion Project in 2021 (202102310570) and Innovative Talents of Philosophy and Social Sciences in Henan Province (2022-CXRC-12).

## Conflict of interest

The authors declare that the research was conducted in the absence of any commercial or financial relationships that could be construed as a potential conflict of interest.

## Publisher’s note

All claims expressed in this article are solely those of the authors and do not necessarily represent those of their affiliated organizations, or those of the publisher, the editors and the reviewers. Any product that may be evaluated in this article, or claim that may be made by its manufacturer, is not guaranteed or endorsed by the publisher.
